# Role of Cannabis in the Incidence of Myocardial Infarction: A Review

**DOI:** 10.7759/cureus.11097

**Published:** 2020-10-22

**Authors:** Amit Banerjee, Arohi B Gandhi, Ishan Antony, Josh Alexander, Mohamed Hisbulla, Vishmita Kannichamy, Ifrah Kaleem, Vinayak Mishra, Safeera Khan

**Affiliations:** 1 Internal Medicine, California Institute of Behavioral Neurosciences & Psychology, Fairfield, USA

**Keywords:** cannabis, marijuana, myocardial infarction

## Abstract

Legalizing cannabis use in various states in the United States has caused increased substance abuse, mostly among young people. There are very little data focussing on marijuana use and myocardial infarction (MI) incidence. The objective of the study is to analyze the published papers for cannabis-induced MI and derive a strong relation between cannabis use and MI and understand the pathophysiology. An online search was conducted in PubMed, Google Scholar, and PubMed Central to find relevant publications examining patients who developed MI due to cannabis use. Out of 32 articles that were identified for this review, 17 are case reports, one is a letter to the editor, eight are observational studies, and six are review articles. Many studies have proposed different mechanisms by which cannabis affects the body. Our study shows that marijuana can precipitate MI even if it is used for the first time. Limited data is available to comment on the mortality of patients after cannabis-induced MI. These findings highlight the necessity for public awareness to prevent the ill-effects of cannabis, especially for teenagers and older people.

## Introduction and background

Cannabis is a flowering plant originating from East Asia and subsequently been brought to various other parts of the world. Though cannabis grows naturally, through a hydroponic system and artificial lighting it can be grown indoors [1,2]. Since cannabis can be cultivated indoors and easily disturbed, its popularity may have increased, and it is now the most commonly used illicit drug in the United States. Cannabis or marijuana is the most preferred psychoactive substance after alcohol and tobacco [1,3]. Marijuana refers to the product made from leaves, seeds, stem, or flowers of the cannabis plant. 

Almost 33 states have legalized cannabis consumption in the United States. There was an estimate of 188 million cannabis users in 2017 [4]. A survey among adolescent groups revealed that about three-fourths of adolescents thought there was no significant harm in cannabis usage [5]. Furthermore, a longitudinal study showed that black youths use it more compared to whites [6]. The use of marijuana is becoming popular among the elderly population as a form of treatment to improve sleep quality and for chronic illnesses. Since the decriminalization of usage, possession, and selling of marijuana, there are various synthetic substances produced and sold commercially with tetrahydrocannabinol (THC) as the main ingredient. Various brand names of synthetic marijuana available on the internet are Armageddon, Aztec Gold, K2, Black Mamba, Cloud-9, Demon, Mad Hatter, and Spice [7]. Azofeifa et al. conducted a study that presented an increase of marijuana consumption by 455% among adults ages 55 to 64 and a 333% increase in those greater than 65 years of age [8]. Vaping of marijuana has gained recent popularity, especially among high school students. It was believed earlier that vaping was safer than smoking because of no carbon monoxide component from heating. It also produced increased blood THC level and psychoactive effect, but recent reports say it causes more damage to the lungs [7,9]. The active chemical present in cannabis is tetrahydrocannabinol (THC).

Cardiovascular disease is the leading cause of death in the United States. It includes arrhythmias, congestive heart failure (CHF), hypertension, coronary artery disease (CAD), peripheral vascular disease (PVD), strokes, and myocardial infarction (MI) [10]. Obesity and cigarette smoking are the major modifiable risk factors for CVD. The documented effects of marijuana on the cardiovascular effects show an increase in heart rate from 20% - 100% after smoking, starting within 10 - 30 minutes. It also reduces the systemic blood pressure, causes orthostatic hypotension, and some forms of arrhythmias like an atrial flutter, atrial fibrillation, and ventricular tachycardia [10].

After the legalization of cannabis in various states in the United States, there is an increasing need to understand and educate the public regarding health hazards, especially on the cardiovascular system. Hence, in this review, we discussed the potential pathophysiological mechanisms that may cause serious cardiovascular effects in cannabis users. Also, we wanted to find the relation between marijuana use and myocardial infarction incidence.

## Review

Methods

We conducted a thorough literature search using PubMed Central (PMC), PubMed, MEDLINE, and Google Scholar. We used Keywords such as 'Myocardial Infarction,' 'Cannabis', and 'Marijuana'. These keywords were used individually and in combination to gather relevant articles. Medical Subject Heading (MeSH) search terms' Cannabis' and 'Myocardial Infarction' were used to collect data. Type of studies included were randomized controlled trials, letters to the editor, review articles, observational studies, and case reports. The results of the keyword searches are summarized in Table 1*.*

**Table 1:** Results of keyword searches

Keywords	PubMed	PubMed Central	Google Scholar
Cannabis	5,474	31,461	723,000
Marijuana	8,531	52,886	994,000
Myocardial Infarction	39,201	273,499	2,510,000
Cannabis and Myocardial Infarction	41	1,267	8,440
Marijuana and Infarction	68	1,913	12,800

Inclusion and Exclusion Criteria

We included relevant articles from the past 12 years, specifically from 2008 to 2020. Papers published in the English language were included. Various sample sizes in the different geographical areas were included in all the papers collected for the study. All animal studies were excluded, and only human studies were included. Furthermore, studies that focused on the medicinal aspect of cannabis were not included. After applying specific inclusion and exclusion criteria, 32 scientific papers were selected for our final review, which met the quality specifications and was peer-reviewed.

Result

The primary screening process identified 172 articles after typing in the keywords in the electronic databases. After applying inclusion and exclusion criteria, 63 articles were shortlisted. After further manual scrutinization, we identified 32 articles suitable for this review. Our article included six review articles, 17 case reports, eight observational studies, and one letter to the editor. All the papers included patients exposed to marijuana and have had at least one episode of a cardiovascular event.

Discussion

Three naturally growing strains of cannabis (sativa, ruderalis, and indica) have been identified as of now. However, interbreeding produced many "hybrid" strains. Marijuana, a by-product of cannabis, has almost 60 chemical products, but the major contributor to the biological effects is the compound tetrahydrocannabinol (THC). The difference in these strains is the content and proportion of the active ingredients THC and cannabidiol (CBD) [3,11]. Illegal synthetic cannabinoids and cannabimimetics are also mass-produced for recreational purposes. They are more popular because of their higher potency, and because conventional drug screening tests do not detect them. These substances include a family of almost 700 synthetic compounds. They are made by altering the chemical properties of THC to raise the affinity towards cannabinoid receptors, augment the duration of action, and increase the downstream signal transduction [3,12]. THC acts on cannabinoid receptor-1 (CB1R) and cannabinoid receptor-2 (CB2R). CB1R receptors are located in the heart, brain, vascular smooth muscle cells, and liver, whereas CB2R receptors are on the immune cells [11]. Smoking marijuana exposes a person to gaseous material and particulates that come from the combustion of plant products. Marijuana affects the cardiovascular system, cerebrovascular system, hepatocellular system, and many others. Here we will discuss how marijuana alters our body's normal physiology and the mechanisms involved in it by reviewing the published articles. We will also find a relation between marijuana use and the incidence of myocardial infarction by analyzing various published case reports and observational studies. 

Mechanism of Action of Cannabis on the Cannabinoid Receptors

The endocannabinoid system (ECS) comprises 2-arachidonylglycerol and endocannabinoids anandamide and their metabolic enzymes and two receptors. CB1R and CB2R are the two receptors that mediate the effects of marijuana [13]. CB1R receptors are in the heart, brain, vascular smooth muscle cells, and liver, whereas CB2R receptors are on the spleen and immune cells [11]. CB1R is the central receptor to facilitate the action of marijuana. Since these receptors are present in the body extensively, its activation affects multiple systems [7]. The molecular mechanism of the receptors involves signal transduction through modulation of adenylyl cyclase (AC), nuclear factor kappa light chain enhancer of activated B cells (NF-kb), and mitogen-activated protein kinases (MAPK) [7,14]. CB1R stimulation causes activation of the adenylyl cyclase inhibitor subunit of G-proteins (Gi/o). This reduces cyclic adenosine monophosphate (cAMP) formation [15]. This, in turn, causes G-protein coupled inwardly rectifying potassium channels (GIRK) to be activated, and also it inhibits the N-type calcium channels [16]. CB1R stimulation also activates MAPK signaling pathways, including c-Jun N-terminal kinase (JNK), extracellular signal-regulated kinase ½ (ERk ½), and p38, which are involved in cell cycle control, cell proliferation, and cell death. CB1R also activates the phosphatidylinositol-3-kinase (PI3K)/protein kinase B (Akt) pathway, regulating cell growth and survival. CB1R can also signal through G-protein-independent mechanisms. It can associate with ß-arrestin, which plays a vital role in G-protein coupled receptor (GPCR) desensitization. It binds to the receptor and starts the internalization process [16]. Intracellular CB1R increases intracellular calcium by releasing calcium from internal lysosomal calcium stores [17]. In the mitochondria, CB1R activation can cause the cAMP formation and reduction in mitochondrial respiration, affecting cellular energy metabolisms [18]. Because of the extensive nature of CB1R signaling, there are many outcomes of CB1R activation. It can lead to cell survival or cell death based on which part of the body it is located in. A great deal is studied about CB1R signaling, whereas there is less understanding of CB2R signaling's role.

Effect of Cannabis on the Myocardium

The myocardial effects of cannabis are due to changes in the coronary blood flow and heart rates, promoting ischemia leading to myocardial infarction [19]. Marijuana consumption results in the activation of the sympathetic autonomic nervous system and inhibition of the parasympathetic autonomic nervous system [20]. This eventually leads to a sudden rise in heart rate, lasting more than an hour after consumption [21]. Acute exposure to marijuana will increase blood pressure and can also trigger atrial fibrillation [22]. This cumulative effect of marijuana on the autonomic nervous system results in increased myocardial demand and cardiac workload. The cardiac output rises by 4% to 9% following marijuana use. Smoking marijuana can also result in a rise of carboxyhemoglobin in the blood due to combustion. Oxygen delivery to all the vital organs is compromised when there is an increase in blood carboxyhemoglobin levels. The red blood cells' capacity to carry oxygen is significantly reduced when there is a high carboxyhemoglobin level. This combination of decreased oxygen supply because of high carboxyhemoglobin levels and an increased myocardial oxygen demand because of tachycardia will create a mismatch in the supply and demand of oxygen, ultimately resulting in transient myocardial ischemia [10].

According to earlier studies, CB1R and CB2R are involved in modulating inflammatory processes in the body. Activation of CB1R results in atherosclerotic and inflammatory effects, whereas activation of CB2R leads to immunosuppression and anti-inflammatory effect. CB1R and CB2R mediate downstream signaling at the cellular level by activating their respective ligands at the cellular level [23]. There are ongoing researches done in the area of immunomodulation by the receptors (CB1R and CB2R). Furthermore, few reviews have outlined that the downstream effects of CB receptor activation possibly lead to systemic pro-inflammatory states [24]. It is postulated that there is an increase in tumor necrosis factor α (TNFα) and interleukin (IL)-12 levels from CB-induced activation of G-protein coupled receptor 55 (GPR55); this, in turn, increases the endocytic function of the monocytes. This can eventually lead to atherosclerosis and foam cell production, which can precipitate MI in the long run [24].

CB1R and CB2R receptors are found on the platelet surface, which are the primary sites for activating platelet aggregation. In animal models, it is found that THC increases cyclooxygenase-1 and cyclooxygenase-2 (COX-1, COX-2) activity leading to thromboxane A2 and many prostaglandin productions [25]. It is shown that THC acting on CB receptors leads to increased platelet aggregation through increased platelet P-selectin and glycoprotein IIa/IIIb expression. Furthermore, THC leads to the formation of 2-arachidonoylglycerol (2-AG), which is a precursor for arachidonic acid [26]. Initially, the effects of 2-AG on the aggregation of platelets start with Phosphatidylinositol 3 Kinase/AKT pathway. This pathway leads to conformational changes in platelet structure by myosin light chain kinase phosphorylation and actin polymerization. This conformational change results in the secretion of adenosine triphosphate (ATP) and platelet aggregation [27]. This increased arachidonic acid production and COX activity result in a pro-inflammatory state in the human body. It leads to vascular endothelial damage, central and peripheral vasoconstriction, and importantly platelet aggregation, which collectively increases the risk of atherosclerosis and other cardiovascular events, including myocardial infarction. If more research is done to understand better the complex mechanisms of platelet aggregation and inflammatory effects of cannabis on the cannabinoid receptors, many serum markers and risks factor for cardiovascular disease like myocardial infarction due to cannabis abuse can be determined to help the increasing cannabis users in the nation.

Effect of Marijuana on Other Systems

Apart from the cardiovascular system, cannabinoid exerts its effects on other systems, leading to CVD eventually. Cannabinoids can also cause psychological disturbances. It causes anxiety, also impairs memory, attention, and psychomotor performance when under the influence. Cannabis abuse may be a reason for poor school performance [28]. In the cerebrovascular system, it can cause cerebral artery luminal stenosis, reversible cerebral vasoconstriction syndrome, and cerebral auto-dysregulation [29]. High-calorie intake and cannabinoid consumption lead to obesity and its metabolic derangements, like diabetes mellitus, sleep disorders, chronic kidney disease, and CVD. The activation of CB1R by the cannabinoids results in increased lipogenesis decreased insulin responsiveness, and defective secretion of insulin from the pancreas, skeletal muscles, liver, and adipose tissues [30]. These altogether can increase endothelial dysfunction. It is proved that short term activation of CB1R causes a rise in appetite, increased intake of food, and body weight and glucose intolerance in healthy young males [30]. This can lead to atherosclerosis, which can precipitate a MI.

Incidence of MI in Marijuana Users

We reviewed the results of different case reports and observational studies to find a stable relationship between cannabis abuse and myocardial infarction onset and the patients' mortality after cannabis-induced myocardial infarction. Many case reports have been published that show patients getting a MI a few hours after marijuana use. In a case report by Kariyanna et al., a 64-year-old African American man experienced retrosternal chest pain lasting for 30 mins following smoking a blunt of marijuana. There was an elevation of ST-segment in electrocardiogram (EKG) leads II, III, and aVF and ST-segment depression in leads of aVL, V4, and V5. They diagnosed it as ST-segment Elevation Myocardial Infarction (STEMI) [31]. Yurtdaş et al. reported a case of a 26-year-old male who came to their clinic with complaints of radiating chest pain to both his upper limbs. He smoked cannabis products two times a week for eight years. EKG showed ST elevation [32]. Zaleta et al. reported a case of an adolescent boy 14 years of age who presented with complaints of sudden onset central chest pain and headache. The boy smoked K2, a synthetic cannabinoid, for the first time, four hours prior to the onset of the symptoms. The examination showed tachycardia and elevated systolic and diastolic blood pressure. His troponin levels were high, and EKG showed ST-segment elevation. A diagnosis of STEMI was made. This indicates that marijuana can induce acute myocardial infarction (AMI) in all ages, from adolescence to old age [33]. Other case reports are shown in Table 2.

**Table 2:** An overview of published case reports describing patients with myocardial infarction associated with marijuana smoking

AUTHOR	AGE/ SEX	TIME FROM MARIJUANA CONSUMPTION TO SYMPTOMS	SYMPTOMS	ECG	TROPONIN LEVELS	RISK FACTORS	THROMBUS LOCALIZATION	TREATMENT
Ul Haq E et al. [28]	31/M	30-40 minutes	Left side radiating chest pain to the left arm	Anterolateral ST elevation	Unknown	None	Left anterior descending artery (LAD)	Aspiration thrombectomy
Ul Haq E et al. [28]	47/M	Six hours	Crushing substernal pain	Extensive ST-segment elevation	Unknown	None	Distal LAD and Right Coronary Artery (RCA)	Aspiration thrombectomy
Ul Haq E et al. [28]	26/M	Few hours	Retrosternal non-radiating chest pain	Lateral ST elevations	Unknown	None	Proximal LAD	Aspiration thrombectomy
Kariyanna PT et al. [31]	64/M	Unknown	Radiating retrosternal chest pain, nausea, Vomiting	ST-segment elevation	26.78 ng/mL	Hypertension, Cigarette smoking	RCA	Medical
Yurtdaş M et al. [32]	26/M	Three hours	Retrosternal chest pain radiating to both arms	ST-segment elevation	34 ng/mL	Cigarette smoking	Proximal RCA	Stents Implanted and Medical
Zaleta S et al. [33]	14/M	Four hours	Sudden onset chest pain, Headache	ST-elevation	32 ng/L	None	Not determined	Medical
Köklü E et al. [34]	31/M	45 minutes	Syncope, sudden cardiac arrest	ST-elevation	Unknown	None	RCA	Stents Implanted and Medical
Keskin M et al. [35]	15/M	Two hours	Chest pain	Right bundle branch block and ST elevation	6.4 ng/mL	None	Normal coronary Angiography Findings	Medical
Landa E et al. [36]	42/M	One hour	Non-radiating Chest pain, Diaphoresis, Vomiting	ST depression (NSTEMI)	27.9 ng/ml	None	RCA and Obtuse Marginal Artery (OMA)	The drug-eluting stent placed, and Medical
Patel KH et al. [37]	61/M	Unknown	sharp substernal chest pain, Diaphoresis, Vomiting	tall hyperacute T-waves (NSTEMI)	0.69 mg/dl	vasospastic angina, hyperlipidemia	OMA	Medical
Orsini J et al. [38]	40/M	Unknown	tonic-clonic seizures	ST-segment elevation	8.32 ng/ml	Past medical history was unreliable	Unable to perform	Died
Lawin D et al. [39]	21/M	Two hours	substernal chest tightness radiating to left shoulder	ST-segment elevation	0.23 ng/ml	None	LAD	Thrombus aspiration, Drug-eluting stent implanted and Medical
Canga Yet al. [40]	28/M	Two hours	chest pain	ST-segment elevation	46 ng/ mL	Cigarette smoking	LAD	Aspiration thrombectomy and Medical
McKeever RG et al. [41]	16/M	60-90 mins	substernal pain with dyspnea, nausea, and vomiting	ST-segment elevation	8.29 ng/mL	Cigarette smoking	Normal coronary angioplasty findings	Medical

Apart from these case reports, there are many cohort studies published that provide additional support to the fact that MI's incidence is increased with marijuana usage. In a multicentre trial conducted by Mittleman, he tried to find whether marijuana use is a potential trigger for MI. In this trial, they interviewed 3882 patients with AMI. They found that among the 3882 patients, nine patients reported smoking within one hour, 37 within 24 hours, and 124 patients reported smoking marijuana in the previous year. He showed that marijuana use increases AMI's risk by almost five times within one hour of smoking marijuana and then rapidly decreases after the initial hour [42]. A retrospective study conducted by Desai et al., involving 2,451,933 patients, concluded that the lifetime risk for AMI is increased by up to 8% in marijuana users [43]. In a study cohort conducted by DeFilippis et al., with 2097 patients, of which 125 were marijuana users, they found a significantly higher proportion of MIs in substance abuse patients (64.7% vs. 52.1%) [44].

Furthermore, in a nationwide retrospective cohort study done by Desai et al., consisting of 52,290,927 young patients from age 18 to 39 years, they found that the prevalence of AMI was greater (0.2% vs. 0.1%) in the marijuana users when compared with the non-users [45]. A prospective exploratory study of marijuana use and mortality following MI by Mukamal et al. found out that marijuana use was associated with three times increased mortality following AMI [46]. Likewise, in another prospective study by Frost et al., they followed up MI survivors for 18 years. Their results suggest that there might be increased mortality associated with marijuana use among myocardial infarction survivors [47]. In contrast, two studies say that there is a decrease in in-hospital mortality post-cannabis-induced MI. Desai et al. and Johnson-Sasso et al., in their research, have found a decrease in mortality following cannabis-induced MI [43,48]. All these published observational studies establish the fact that marijuana use increases the risk of myocardial infarction.

Limitations

Our review article had some limitations. One of them was that the study could not focus on any specific age groups. The articles included did not have any randomised controlled trials (RCT) or meta-analyses. Most of the studies had no follow-up data of the patients who experienced cannabis-induced MI, so the mortality of those patients after an attack of MI could not be determined. Several articles that were included in the study lacked specific risk factors and the personal history of the patients.

## Conclusions

Over the past decade, marijuana use has increased rapidly. Legalizing possession, consumption, and distribution of marijuana in various states of the United States is the prime reason. Our study finds a strong relationship between marijuana use and the incidence of myocardial infarction and mortality of patients after cannabis-induced MI. Many cases in our research show that after marijuana use, even for the first time, there can be an event of MI, indicating that marijuana use should be considered a significant risk for MI. Mortality of patients after cannabis-induced MI could not be determined effectively due to insufficient data, but provided data says that there is a decrease in in-hospital mortality post-cannabis-induced MI. Various studies have proposed the pathophysiology of how these events occur. It is safe to say that cannabinoids act on the cannabinoid receptors to affect the cardiovascular system. They cause a mismatch in oxygen supply and demand in the myocardium, which can lead to ischemia. It can also increase platelet aggregation, which can lead to atherosclerosis, ultimately MI. The majority of the public use this for recreational purposes, thinking it is a safe drug, especially teenagers and older people. Public awareness about the ill-effects of marijuana is the need of the hour, and all physicians should always recognize those effects and advise their patients properly.
